# Examination of Motor Imagery Ability in Individuals With Diplegic Cerebral Palsy: Case-Control Study

**DOI:** 10.31083/RN37324

**Published:** 2025-11-27

**Authors:** Dilan Demirtas Karaoba, Gulfem Ezgi Ozaltin, Busra Candiri, Burcu Talu

**Affiliations:** ^1^Physiotherapy and Rehabilitation Department, Faculty of Health Sciences, Igdir University, 76000 Igdir, Türkiye; ^2^Physiotherapy and Rehabilitation Department, Faculty of Health Sciences, Inonu University, 44000 Malatya, Türkiye

**Keywords:** cerebral palsy, imagery, reaction time, spastic diplegia, parálisis cerebral, imaginería, tiempo de reacción, diplejía espástica

## Abstract

**Introduction:**

Given the important role of motor imagery (MI) in rehabilitation, this study aimed to compare MI abilities in individuals with spastic diplegic cerebral palsy (SDCP) and typically-developing (TD), and to determine the factors associated with MI ability in SDCP.

**Patients and Methods:**

This study was planned as a cross-sectional, case-control study. SDCP (n = 26) and TD (n = 26) individuals participated in the study. SDCP individuals were selected from Special Education and Rehabilitation Centers, while TD participants were recruited from relatives of patients receiving therapy at these centers and from volunteers responding to bulletin board announcements. All assessments were performed before or after the weekly physiotherapy sessions, to avoid interfering with routine physiotherapy and rehabilitation sessions. Visual and kinesthetic imagery abilities were assessed using the Movement imagery questionnaire for children (MIQ-C), Implicit MI capacity laterality task, and Explicit MI capacity mental chronometry.

**Results:**

The SDCP group had a mean age of 11.69 (3.78) years, consisting of 12 females and 14 males; 10 participants were classified as Gross Motor Function Classification System (GMFCS) Level I and 16 as Level II. The TD group had a mean age of 11.50 (2.30) years, including 16 females and 10 males. A significant difference was found between the groups in MIQ-C and mental chronometry performance (*p* < 0.05). While there was a significant difference in reaction time according to dominance in SDCP (*p* = 0.038), there was no difference in accuracy rate (*p* = 0.699). Reaction time and accuracy rate were significantly different between groups according to dominance (*p* < 0.05). There was no correlation between MIQ-C total score, dominant reaction time and accuracy rate and age, Body Mass Index (BMI), and GMFCS (*p* > 0.05). While age and BMI were not related to mental chronometry; GMFCS was found to have a significant positive effect on mental chronometry (*p* = 0.000).

**Conclusions:**

In children with SDCP MI ability differs from that of typically developing peers, being weaker across all assessed subparameters. Moreover, MI ability showed a moderate association with the GMFCS level.

## Abstract (es)

**Introducción:**

Dada la importancia de la imaginería motora (IM) en la rehabilitación, este estudio tuvo como objetivo comparar las capacidades de IM en personas con parálisis cerebral dipléjica espástica (PCD) y personas con desarrollo típico (TD), y determinar los factores asociados con la capacidad de IM en la PCD.

**Pacientes y Métodos:**

Este estudio se planificó como un estudio transversal de casos y controles. Participaron personas con PCD (n = 26) y con TD (n = 26). Las personas con PCD se seleccionaron en Centros de Educación Especial y Rehabilitación, mientras que los participantes con TD se reclutaron entre familiares de pacientes que recibían terapia en estos centros y entre voluntarios que respondían a los anuncios en los tablones de anuncios. Todas las evaluaciones se realizaron antes o después de las sesiones semanales de fisioterapia, para evitar interferir con las sesiones rutinarias de fisioterapia y rehabilitación. Las capacidades de imaginería visual y cinestésica se evaluaron mediante el Cuestionario de Imaginería de Movimiento para Niños (MIQ-C), la tarea de lateralidad de la capacidad de IM implícita y el cronómetro mental de la capacidad de IM explícita.

**Resultados:**

El grupo SDCP tenía una edad media de 11,69 (3,78) años, compuesto por 12 mujeres y 14 hombres; 10 participantes fueron clasificados como sistema de clasificación de la función motora gruesa (GMFCS) Nivel I y 16 como Nivel II. El grupo TD tenía una edad media de 11,50 (2,30) años, incluyendo 16 mujeres y 10 hombres. Se encontró una diferencia significativa entre los grupos en el rendimiento de MIQ-C y cronometría mental (*p* < 0,05). Si bien hubo una diferencia significativa en el tiempo de reacción según la dominancia en Parálisis cerebral dipléjica espástica (PCDE) (*p* = 0,038), no hubo diferencia en la tasa de precisión (*p* = 0,699). El tiempo de reacción y la tasa de precisión fueron significativamente diferentes entre los grupos según la dominancia (*p* < 0,05). No hubo correlación entre la puntuación total de MIQ-C, el tiempo de reacción dominante y la tasa de precisión y la edad, el índice de masa corporal (IMC) y el GMFCS (*p* > 0,05). Si bien la edad y el IMC no se relacionaron con la cronometría mental, se observó que el GMFCS tuvo un efecto positivo significativo en la cronometría mental (*p* = 0,000).

**Conclusiones:**

En los niños con PCDE, la capacidad de MI difiere de la de sus compañeros con desarrollo normal, siendo menor en todos los subparámetros evaluados. Además, la capacidad de MI mostró una asociación moderada con el nivel de GMFCS.

## 1. Introduction

Cerebral palsy (CP) is a permanent but non-progressive developmental disorder that causes dysfunction resulting from a brain lesion experienced before birth, at birth, or during infancy. Spastic diplegic CP (SDCP) is characterized by functional and motor disorders that affect both sides of the body and are more dominant in the lower extremities [1]. Overall, CP affects approximately 2.1 out of every 1000 live births [2]. Spastic CP, which causes widespread motor impairment, represents 85% of the diagnosed population [3] and SDCP represents 35% [4]. Magnetic resonance imaging (MRI) plays a critical role in the diagnosis and prognosis of CP by providing detailed visualization of brain lesions, their laterality, and severity. Advances in MRI technology, including new volume rendering techniques, are improving early diagnosis and characterization of brain injuries even at the fetal stage, providing valuable insight into the extent of neurological impairment and guiding clinical decision making [5]. These children show impairments in visual-spatial and sensorimotor functions, as well as deficits in attention and executive functions [6, 7]. In individuals with CP, the disorder between the visual and proprioceptive systems and changes in their bodily perceptions may cause social perception deficits [8]. It has also been found that many individuals with CP have visual deficits associated with visual field narrowing or oculomotor problems, leading to visual-perceptual impairments [9]. It can be thought that visual-perceptual disorders also affect visual imagery skills in these people.

Motor impairments in individuals with CP cause a lack of motor anticipatory planning, resulting in an impairment in the ability to use motor images, i.e., motor imagery (MI) [10]. MI, which includes the mental process in which the individual mentally imagines that movement without actually performing an active movement, refers to the capacity to produce kinesthetic representations of motor movements [11]. Actively performed motor movements and motor imagined movements occur in the premotor and parietal areas, basal ganglia, and cerebellum [12]. Based on this, MI allows identifying the cognitive and cerebral properties of movement representation independently of motor output and sensory feedback [13]. In the studies carried out, the problems arising from the cognitive process planned before the movement were emphasized and the influence of MI, which is the basic structure of motor simulation, was investigated [14]. Additionally, various studies have shown that MI is used to predict the proprioceptive consequences of movement and support movement planning [15]. Deficits in body and motor representations in children with CP have also been investigated through MI [16, 17]. Various methods have been developed to evaluate people’s MI ability, such as questionnaires, mental chronometry, and choice task [18, 19].

When the literature is examined, it is seen that MI capacity is mainly the focus in hemiplegic CP [10, 14] while these studies are not sufficient in individuals with SDCP [20, 21]. Examining MI through various categories—such as visual, kinesthetic, implicit, and explicit—provides a more holistic assessment of imagery ability. Visual MI refers to imagining the movement from an external perspective (as if watching), whereas kinesthetic MI involves feeling the movement internally [22]. Implicit MI involves automatic imagery, such as during mental rotation tasks, while explicit MI is a conscious and deliberate simulation of movement [17, 23]. Evaluating all these subcomponents helps address the limitations of single-parameter methods used in earlier studies and captures the multifaceted nature of MI. In studies, the latency task or a single parameter was mostly used to evaluate MI capacity, and conflicting results were reported [24, 25]. The small number of studies on MI in individuals with CP is limited, especially in individuals with hemiplegic CP. A recent systematic review reported moderate-quality evidence that individuals with CP have impaired MI skills compared to typically developing individuals. However, only one of the seven included studies focused on bilateral CP, making the results difficult to interpret and indicating the need for further research [26]. Importantly MI ability is closely tied to cognitive and motor development, and age is considered a critical factor influencing MI in children. Younger children may exhibit less developed motor planning, spatial awareness, and working memory—all essential components for accurate MI. Several studies have shown that MI skills progressively improve throughout childhood and early adolescence, in line with the maturation of premotor and parietal brain areas responsible for motor simulation [27, 28]. Molina *et al*. [29] emphasized that MI abilities begin to develop around the age of 7 in typically developing children. In line with this, studies on children with developmental coordination disorder have also typically included participants aged 7 years and older [30, 31]. However, to date, no definitive age threshold for MI development has been clearly identified for children with SDCP. This lack of consensus further underscores the importance of age as a consideration in MI research involving children with neurological conditions. To better understand MI, it has become necessary to investigate different types of CP and examine all aspects of MI. We also believe that investigating the related factors affecting MI in children with SDCP and adding MI training to rehabilitation programs will contribute to treatment programs. This study aims to fill the gap in the literature. It was planned to reveal the difference between MI abilities of individuals with SDCP and typical development (TD) individuals and to determine the factors associated with MI ability in the relevant population.

## 2. Materials and Methods

### 2.1 Study Design and Participants

This study was conducted as a non-invasive, multicenter, cross-sectional case-control study between July 2023 and September 2023. The research was conducted in Special Education and Rehabilitation Centers for individuals with SDCP. Individuals with typical development, were selected from among the relatives of these children in rehabilitation centers, among individuals who saw the voluntary participation announcement posted on the announcement board of the institution and requested voluntary participation. Local ethics committee approval was obtained (Approval number: 23/02, Approval Date: 20/06/2023) and the study was conducted by the Declaration of Helsinki. At the beginning of the study, individuals and their parents signed an informed consent form stating that they would participate voluntarily.

Individuals who were diagnosed with SDCP by a specialist, were between the ages of 7–18, had Gross Motor Function Classification System (GMFCS) I–II, had a cognitive level to follow the instructions given by the researchers, and had an IQ >70 were included. Exclusion criteria; Those with severe hearing, vision, and attention problems, those who have had a seizure in the last 6 months, or those who cannot be controlled despite seizure medications. Since it is known that MI ability begins to emerge at the age of 5–6, children aged 7–18 were included, similar to other studies [18, 32, 33, 34, 35].

Inclusion criteria for TD individuals were being between 7 and 18 years of age, having no history of musculoskeletal problems or neurological disorders, and having no chronic illnesses or cognitive problems.

The population of the research consisted of individuals diagnosed with SDCP and TD individuals in rehabilitation centers. The purpose of the mental stopwatch test is to assess the temporal coherence between real and imagined movement [34]. A power analysis was performed considering the relevance of the timed up-and-go test (TUG) to our primary outcome measure, the mental stopwatch. In the power analysis performed before the start of the study, with α = 0.05 and 1-β (power) = 0.80, It was calculated that at least 23 individuals should be included in each group for the change in TUG score to be 2 [36]. Power analysis was performed using the publicly available statistical software OpenEpi, version 3 (http://www.openepi.com) to calculate the sample size.

Initially, 34 individuals diagnosed with SDCP were screened for eligibility. Of these, 2 were excluded due to cognitive impairments that limited their ability to follow instructions, and 3 were excluded due to experiencing seizures within the past six months. Additionally, 3 participants were excluded at a later stage because they did not complete the assessment procedures. For the TD group, 32 individuals were initially assessed. Six were excluded: 3 due to reported chronic health conditions and 3 due to uncorrected severe visual impairments. Consequently, 26 participants with SDCP and 26 TD individuals were included in the final analyses.

### 2.2 Measures

Data were collected before or after weekly physiotherapy sessions in a way that would not affect the physiotherapy and rehabilitation practices of the children. Data were collected from the participants only once and there were no repetitions of measurements. Participants’ socioeconomic status and the length of time they had been receiving rehabilitation were also recorded. Socioeconomic status (SES) was assessed based on parent-reported information regarding educational attainment and occupational status, which are widely recognized indicators in SES classification. Parental education levels were categorized into three groups: (1) primary school or below, (2) high school graduate, and (3) university degree or higher. Occupational status was also grouped into three categories: (1) unemployed or unskilled labor, (2) semi-skilled/skilled labor, and (3) professional or managerial positions. Based on the combined evaluation of these two variables, participants were classified into low, moderate, or high SES categories, following classification frameworks commonly used in national statistics and previous pediatric rehabilitation research [37, 38]. Rehabilitation history refers to the duration of regular rehabilitation after diagnosis. Participants regularly attended physiotherapy and rehabilitation twice a week. Total treatment duration was recorded in years.

### 2.3 Movement Imagery Questionnaire for Children

Movement imagery questionnaire for children (MIQ-C) was used to assess visual (internal, external) and kinesthetic imagery ability. The questionnaire developed by Martini *et al*. [18] contains 12 items (4 items for internal, 4 items for external, 4 items for kinesthetic imagery ability) [18]. In total, 4 different movements were imaged from 3 different imagery perspectives. The individual imagined the movement after performing the movement once in reality. The clarity of imagery was scored using a Likert-type scale (1: very difficult to feel–7: very easy to feel). Higher scores indicate better mental imagery ability [18].

### 2.4 Laterality Task (Selection Task)

Implicit MI capacity was measured as a laterality task, that is, deciding which side a limb belongs to. Right-left discrimination of the foot was evaluated using the “Recognise Foot” application developed by the Neuro Orthopedic Institute. The “Vanilla” part of the application was used. A total of 20 images of the foot were shown at 5-second intervals, then reaction times and accuracy percentages were recorded [39].

### 2.5 Mental Chronometry Paradigm

A mental chronometry was used to assess explicit MI capacity. This paradigm compares actual movement time with imagined similar task time [19]. Mental chronometry absolute time moving away from 0% indicates that MI ability is more affected [40]. Mental chronometry delta (Δ) time measurements were made for TUG. After the relevant test was performed, individuals imagined the test with their eyes closed. The following formula was used to calculate mental chronometry time [40]. Mental chronometry = Actual movement – imagined movement (Actual movement + imagined movement)/2 × 100.

### 2.6 Statistical Analysis

IBM SPSS Statistics for Windows, Version 25.0 (IBM Corp., Armonk, NY, USA) was used to analyze the data. The suitability of the data for normal distribution was tested with the Shapiro-Wilk test. Descriptive statistics were mean, standard deviation, and median (min-max). Independent Sample *t*-test, Mann-Whitney U test, and Chi-Square test were used to compare the basic variables and MI ability of the SDCP and TD groups between the groups. Paired Sample *t*-test and Wilcoxon signed-rank test were preferred to compare the laterality task according to dominance within the group. Multiple linear regression analysis was used to determine the relationships between mental chronometry performance and age, Body Mass Index (BMI), and Gross Motor Function Classification System (GMFCS), with MIQ-C total score, right dominant reaction time, and accuracy rate as dependent variables. A significance level of *p* < 0.05 was considered statistically significant.

## 3. Results

A total of 52 people in the SDCP and TD groups participated in the study. There was no significant difference between the groups in terms of age, BMI, and gender (*p* > 0.05). The basic variables of the groups are listed in Table 1.

**Table 1.** **Baseline characteristics of CP and control group**.

		CP group (n = 26)	Control group (n = 26)	*p*
Age, year		11.69 (3.78)	11.50 (2.30)	0.618^a^
BMI, kg/m^2^		19.54 (3.38)	19.10 (2.65)	0.597^b^
Gender (F/M)		12/14	16/10	0.266^c^
Socioeconomic status (n, %)				
	Low	19 (73.1)		
	Moderate	7 (26.9)		
	High	-		
Rehabilitation history, year		8.50 (3.60)		
GMFCS (I/II)		10/16		

Mean (SD), ^a^Mann Whitney U test, ^b^Independent Sample *t*-test, ^c^Chi-Square test. CP, Cerebral Palsy; BMI, Body Mass Index; F, Female; M, Male; GMFCS, Gross Motor Function Classification System.

A significant difference was found between the SDCP and TD groups in terms of MIQ-C subsections total scores and mental chronometry performance (*p* < 0.05) (Table 2).

**Table 2.** **Within and between groups analysis of recognition accuracy and reaction time**.

		CP group		*p*	Control group		*p*	*p* between groups on dominant hand	*p* between groups on non-dominant hand
		Dominant	Non-dominant		Dominant	Non-dominant			
Laterality task	Reaction time, s	2.74 (0.51)	2.97 (0.43)	0.038^c^	2.30 (0.56)	2.35 (0.49)	0.640^c^	0.006^a^	0.000^a^
	Accuracy, %	48.84 (15.83)	50.00 (16.97)	0.699^c^	80.00 (15.74)	79.23 (16.71)	0.788^d^	0.000^b^	0.000^b^
Movement imagery questionnaire for children	MIQ-C, internal	5.50 (2.25–7)			6.87 (4–7)		0.012^a^		
	MIQ-C, external	5.62 (2–7)			6.12 (2.75–7)		0.007^a^		
	MIQ-C, kinaesthetic	5.50 (2.50–7)			6.25 (3.50–7)		0.011^a^		
	MIQ-C, total	17.12 (7.50–21)			19.25 (11.50–21)		0.008^a^		
Mental chronometry paradigm	mental chronometry Δ	10.15 (2.73–20)			2.11 (0.15–4.68)		0.000^b^		

^a^Independent Sample *t*-test, ^b^Mann-Whitney U test, ^c^Paired Sample *t*-test, ^d^Wilcoxon signed-rank test. Δ, Delta; CP, Cerebral palsy; MIQ-C, movement imagery questionnaire for children. Mean (SD), *p* < 0.05.

A significant difference was found in reaction time according to dominance in the SDCP group (*p* = 0.038). There was no significant difference in terms of accuracy rate (*p* = 0.699). There was no difference in reaction time and accuracy in the TD group according to dominance (*p* > 0.05). Reaction time and accuracy rate were significantly different between the groups according to right and left extremity dominance (*p* < 0.05) (Table 2).

The regression model for MIQ-C total, dominant reaction time, and dominant accuracy had low or negative adjusted R^2^ values (Adjusted R^2^ = –0.038; R^2^ = 0.067; R^2^ = –0.070, respectively). No significant variables were found for age, BMI, socioeconomic status, rehabilitation history, and GMFCS level in these models (*p* > 0.05). This indicates that the model could not explain the changes in MIQ-C, dominant reaction time, and dominant accuracy. On the other hand, these parameters together explained 44.4% of the total variance in mental chronometry performance (Adjusted R^2^ = 0.444). In this model, only GMFCS level showed a significant and positive effect on mental chronometry (*p* < 0.001). Other variables were not significant (*p* > 0.05) (Table 3).

**Table 3.** **Regression analysis for factors affecting motor imagery ability**.

	MIQ-C total (adjusted R^2^ = –0.038)				Dominant reaction time (adjusted R^2^ = 0.067)				Dominant accuracy (adjusted R^2^ = –0.070)				mental chronometry (adjusted R^2^ = 0.444)			
	B	S.E.	Beta	*p*-value	B	S.E.	Beta	*p*-value	B	S.E.	Beta	*p*-value	B	S.E.	Beta	*p*-value
Constant	20.895	6.995		0.007	1.996	0.758		0.016	34.133	24.727		0.183	–1.120	5.425		0.839
Age	0.672	0.960	0.559	0.492	0.047	0.104	0.340	0.658	–1.903	3.393	–0.455	0.581	–1.149	0.744	–0.902	0.138
BMI	–0.520	0.381	–0.387	0.187	0.074	0.041	0.483	0.087	–0.311	1.346	–0.666	0.820	0.053	0.295	0.037	0.859
Socioeconomic Status	2.329	2.138	0.232	0.289	–0.412	0.232	–0.359	0.090	4.957	7.558	0.142	0.519	1.295	1.658	0.122	0.444
Rehabilitation history	–0.682	1.000	–0.540	0.503	–0.063	0.108	–0.438	0.567	3.553	3.536	0.809	0.327	1.209	0.776	0.904	0.135
GMFCS	0.200	1.948	0.022	0.919	–0.064	0.211	–0.061	0.766	1.008	6.887	0.032	0.885	6.773	1.511	0.698	<0.001

S.E., Standard Error; BMI, Body Mass Index; GMFCS, Gross Motor Function Classification System.

## 4. Discussion

In this study, the MI ability of SDCP and TD individuals was evaluated in all aspects including implicit, explicit and visual internal, visual external and kinesthetic dimensions. There were differences in all subparameters of MI between individuals with SDCP and TD individuals. At the same time, this study showed that there was a significant relationship between the functional motor level and explicit motor imagery ability of individuals with SDCP.

CP presents symptoms with sensory-motor integration disorders, activity limitations, and motor dysfunction [41]. The underlying cause of the motor disorder has not been adequately explained by spasticity, loss of strength, and abnormal motor synergies. There is an impairment in high-level sensory-motor integration [42]. Movement and perception are two factors that go together and mutually influence each other. In this process, perception is an active process that accompanies the execution of the sensory information required before the movement is carried out with the planned motor program [43]. Children with CP experience deficits in planning the movement as well as performing the movement. Before performing the movement, the mental process comes into play. This process is a complex process that includes preparation, planning, and coordination of movement [44]. MI takes an active role during the mental representation of movement.

MI is a mental process created implicitly or explicitly for the representation of movement, without a real motor movement. Implicit MI requires the individual to make predictions about the movement that will occur by using visual stimuli for movement [26]. Implicit MI is often assessed with hand laterality judgment tasks. The majority of studies on MI so far have been conducted in individuals with unilateral CP (right hemiplegic CP). Considering that motor planning skills are more dominant in the left hemisphere, it is correct to evaluate this sample [45]. However, the view that motor planning and motor execution skills originate not only from the affected hemisphere but also from impaired sensory-motor integration explains why our study was conducted in individuals with SDCP [46].

In our study, there was a significant difference in the results of implicit MI between individuals with SDCP and TD. Individuals with SDCP showed longer reaction times and significantly lower percent accuracy. MI studies have been mostly conducted on unilateral CP so far. There is moderate evidence that individuals with unilateral CP and TD individuals show similar reaction times in implicit MI ability and individuals with unilateral CP show lower accuracy [47, 48]. This situation is generally stated as left hemisphere involvement, where motor planning skills are mostly carried out. During MI, there is an increase in activation of the posterior parietal cortex. This may be an indicator of decreased MI ability in children with SDCP [49]. However, somatosensory body representation and lack of experience in the use of the lower extremities may have limited the recognition ability [50]. In our study, there was a difference in reaction time between dominant and non-dominant hands in the SDCP group in intra-group evaluations, there was no difference in accuracy. This may reflect the biomechanical advantage of the dominant side limb. At the same time, hemisphere roles cannot be kept separate from each other for both sides of the body during the planning phase of motor behavior. Because both sides are moved together in a purposeful movement [51]. Therefore, we can say that MI ability is also weak in children with SDCP who do not have unilateral hemisphere involvement. The laterality judgment test is stated as the test that provides effective evidence about implicit MI [45]. However, more targeted and explicit approaches are needed to better understand MI ability in children with CP.

Explicit MI is the conscious, mental evaluation of an action [26]. In our study, explicit MI was evaluated with the mental chronometry paradigm. TUG test was used for evaluation. There was a significant difference in ΔTUG values ​​between SDCP and TD individuals. Our results are consistent with the literature. Molina *et al*. [21] SDCP used a walking task (Mental chronometry paradigm) to compare explicit MI ability between hemiplegic CP and TD individuals. As a result of the study, it was shown that as the walking distance of individuals with SDCP increased, the MI ability decreased compared to TD individuals. According to these results, MI ability may show different results in different conditions [21]. The TUG test we used for the mental chronometry paradigm in our study has more complex task characteristics as it includes activities such as getting up from sitting, walking, turning back, and coming to the starting point, in addition to walking straight. This allowed us to better understand and evaluate the MI ability of children with SDCP. The factors affecting motor imagery ability were investigated in the present study. According to the regression analysis, it was revealed that the GMFCS level of the children had a significant effect on the mental chronometry paradigm. This situation suggests that timing skills are negatively affected as the level of motor limitations increases. Age, BMI, socioeconomic status and previous rehabilitation history were not significant predictors of MI ability. However, although socioeconomic status was not significantly different, the relationship between reaction time and rehabilitation history was striking. Reaction time may decrease as socioeconomic status increases. These factors may yield significant results in a larger sample group. In the present study, three of the four regression models (MIQ-C total, dominant reaction time, and dominant accuracy) showed very low or even negative adjusted R^2^ values, indicating that the included predictors explained only a small portion of the variance in these motor imagery outcomes. This suggests that other unmeasured factors may have a considerable influence on MI ability in children with SDCP. Possible contributors include cognitive functions closely related to MI, such as working memory, attention, and executive control [52, 53]; developmental differences in motor planning and cognitive maturation during childhood and adolescence; and neuroplastic changes associated with these stages [28, 52]. Psychological factors (e.g., motivation, engagement, familiarity with imagery tasks) and environmental factors (e.g., parental support, access to therapy, and home practice routines) may also play a role [54]. The low explanatory power found here highlights the importance of including these cognitive, psychological, and environmental variables in future predictive models, ideally in larger and age-stratified samples, to better capture the complexity of MI skills in children with CP.

Importantly, these findings also provide insight into potential clinical applications. The strong association between GMFCS level and mental chronometry performance highlights the need to consider functional motor level when designing motor imagery-based rehabilitation strategies. In Special Education and Rehabilitation Centers, MI training protocols could be tailored based on the individuals GMFCS classification. For example, individuals with higher functional levels might benefit from more dynamic and self-directed imagery tasks, whereas individuals with greater motor limitations may require more structured, therapist-guided MI interventions that include external visual cues and simplified motor scenarios. By aligning MI interventions with the functional status of the individual, rehabilitation programs can more effectively target the motor planning deficits specific to this population and enhance motor learning potential.

Visual internal visual external and kinesthetic MI results were found to be lower in individuals with SDCP than in those with TD. While individuals with SDCP showed similar visual internal and kinesthetic features within the group, it was observed that their visual external ability was better. The kinesthetic component refers to body movements and positioning of the body, as well as how the movement is felt. The visual component refers to the internal (first-person perspective) and external (third-person perspective) representation of what the child sees. External perspective represents the child seeing himself as if he were watching himself on television [18]. In our study, external MI ability was higher in individuals with SDCP than internal MI; Sensory-motor involvement may be associated with the more comprehensible visualization ability of these children using a third-person perspective.

The individuals participating in the study could have been classified according to age groups and the effect of age could have been better understood, but the fact that we could not capture the same density in each age group can be shown as a limitation of this study. Another important limitation of the current study is the lack of detailed intelligence assessments. Although all participants had IQ scores above 70, additional cognitive domains such as working memory, attention, and executive functions were not assessed. Although it has been reported that including individuals with IQs below 70 caused distortions in the assessment of MI ability [55], it has also been shown that intelligence does not affect MI ability in individuals with unilateral CP [47]. The contradictory results emphasize the need for further studies investigating the effect of intelligence on MI ability. Another limitation was that the physical activity levels of the individuals were not assessed. Since subjective assessments of physical activity such as questionnaires are based on self-reporting, they are not suitable for children and should be confirmed with objective methods such as pedometers. For objective methods, observation over a period of time is required [56]. For these reasons, the cross-sectional design of the study limited the detailed assessment of physical activity. Future studies may benefit from long-term follow-up of physical activity levels and their relationship with MI ability. Although age did not emerge as a significant predictor in our regression models, this may partly be due to the broad age range of participants (7–18 years), which could have diluted age-related effects. Age is a critical factor for MI performance because motor planning, cognitive control, and the integration of sensory feedback undergo substantial refinement during childhood and adolescence, supported by ongoing neuroplasticity. For instance, previous studies have shown that younger children often demonstrate less efficient motor planning and slower imagery–execution coupling compared to older peers, reflecting the gradual maturation of related neural networks [27, 57]. It is therefore plausible that developmental stage differences within our cohort may have influenced MI outcomes, but the wide age range and uneven distribution across age groups limited our ability to detect these effects. Future studies may benefit from recruiting age-stratified samples and explicitly examining developmental trajectories of MI ability in CP.

A major interest of the study was the examination of all aspects of motor imagery ability. Studies to date have investigated MI ability in children with hemiplegic CP. To our knowledge, our study is the first to investigate all aspects of MI ability (implicit, explicit, visual, and kinesthetic) in children with SDCP. Although there are different treatment options for individuals with brain damage in the literatüre [58, 59, 60], we believe that the findings of this study will illuminate rehabilitation programs to be developed in the future.

## 5. Conclusions

The present study showed that all aspects of MI ability were poorer in individuals with SDCP than in those with TD. At the same time, although age and BMI did not have a direct effect on MI ability, a moderate effect of GMFCS level on MI ability was observed. Understanding MI ability in individuals with SDCP will help identify the weaknesses and strengths of these individuals. Accordingly, intervention strategies can be developed.

## Disclosure

This study was accepted as an oral presentation to the 8. International Hippocrates Congress on Medical and Health Sciences (March 04–05, 2022), and Internatıonal Maldıa Health Scıences Congress (October 14–15, 2022) (also online).

## Availability of Data and Materials

The datasets generated and analyzed during the current study are available from the corresponding author on reasonable request. Due to ethical restrictions and to protect participant confidentiality, the data cannot be publicly shared.
